# The effect of noise on the cortical activity patterns of speech processing in adults with single-sided deafness

**DOI:** 10.3389/fneur.2023.1054105

**Published:** 2023-03-16

**Authors:** Ji-Hye Han, Jihyun Lee, Hyo-Jeong Lee

**Affiliations:** ^1^Laboratory of Brain and Cognitive Sciences for Convergence Medicine, Hallym University College of Medicine, Anyang, Republic of Korea; ^2^Ear and Interaction Center, Doheun Institute for Digital Innovation in Medicine (D.I.D.I.M.), Hallym University Medical Center, Anyang, Republic of Korea; ^3^Department of Otorhinolaryngology-Head and Neck Surgery, Hallym University College of Medicine, Chuncheon, Republic of Korea

**Keywords:** single-sided deafness (SSD), speech-in-noise processing, sound localization, hemispheric lateralization, auditory cortical activation

## Abstract

The most common complaint in people with single-sided deafness (SSD) is difficulty in understanding speech in a noisy environment. Moreover, the neural mechanism of speech-in-noise (SiN) perception in SSD individuals is still poorly understood. In this study, we measured the cortical activity in SSD participants during a SiN task to compare with a speech-in-quiet (SiQ) task. Dipole source analysis revealed left hemispheric dominance in both left- and right-sided SSD group. Contrary to SiN listening, this hemispheric difference was not found during SiQ listening in either group. In addition, cortical activation in the right-sided SSD individuals was independent of the location of sound whereas activation sites in the left-sided SSD group were altered by the sound location. Examining the neural-behavioral relationship revealed that N1 activation is associated with the duration of deafness and the SiN perception ability of individuals with SSD. Our findings indicate that SiN listening is processed differently in the brains of left and right SSD individuals.

## Introduction

One very common concern in individuals with single-sided deafness (SSD) is difficulty following a conversation in a noisy environment such as in classrooms and cocktail party situations. The difficulty arises due to limited accessibility to interaural cues, including the interaural time difference and the interaural level difference (1). Furthermore, this perceptual difficulty worsens with an increase in the duration or severity of hearing loss (2, 3). Nonetheless, conventional hearing-assistive devices, including bone-conduction and contralateral-routing-of-signals (CROS) hearing aids that aim to increase hearing thresholds in the auditory periphery, have shown very limited efficacy in overcoming listening difficulty (4, 5). These findings have led to the hypothesis that cortical elements such as the degree of cortical plasticity or the efficiency of neural transmission may significantly affect perceiving specific sounds in noise. Although concern over this phenomenon is widespread, there is a paucity of published studies in which researchers attempted to directly relate listening difficulty in noise to neural function in individuals with SSD.

Comprehending speech-in-noise (SiN) is a complex task involving both the auditory cortex and many other cortical regions, as evidenced by numerous neuroimaging studies (6–9). This could be because listening to and making sense of speech involves multiple steps of neural processing, including stimulus encoding, selective attention, and working memory. The multi-faceted neural processes involved in SiN perception assessed using various types of measurements such as behavioral tests (10), electrophysiology (11), and functional magnetic resonance imaging (fMRI) (12) have been applied to measure cortical processes during SiN listening. Evidence from previous studies indicates that sensory encoding in both peripheral and higher levels of cortical functioning contribute to SiN perception. For example, in listeners with normal hearing (NH), cortical alpha rhythms are related to digit-in-noise identification performance (13, 14) and those who had earlier subcortical responses reveal better SiN perception (15). These outcomes indicate that SiN listening stimulates different neural processing mechanisms from speech-in-quiet (SiQ) situations and the presence of noise alters the patterns of hemispheric lateralization in both the cortical and subcortical structures of the auditory system (16).

SiN perception is closely related to how an acoustic signal is transmitted along and transformed by the central auditory system. Given that introducing noise can disrupt signal encoding in the auditory system, noise that interferes with a signal is often referred to as a masker. Electroencephalography (EEG) has been applied to study effects of a masker on speech processing since it is sensitive to subtle neural changes and has excellent temporal resolution. Among the EEG components, it has been shown that the fidelity of N1/P2 is capable of predicting SiN performance in various populations, such as cochlear implant (CI) users and children with learning disorders (17–19). For instance, CI users revealed decreased N1 amplitude and delayed P2 latency in response to SiN listening, while the cortical responses are significantly associated with behavioral SiN measures (18). Neural responses in simulated unilateral CI users are temporally delayed for noise-vocoded speech tasks (20). Meanwhile, the patterns of hemispheric lateralization during SiN listening in individuals with SSD differ from those in NH people in that the alpha and theta neural activities are left-lateralized in the latter but greater toward the direction of the background noise in the former (21). Although the cortical processes in populations with hearing impairments during SiN listening have been investigated in recent years, only a few researchers have observed relationships between neural function and behavioral SiN performance in people with SSD. Hence, a more systematic approach to providing insight into the brain mechanism underlying SiN perception is needed.

Since spatial hearing is dependent on information based on the interaural acoustic difference and spectral cues, it is important for listening in a noisy environment as well. Moreover, unilateral hearing loss can incur deficits not only in behavioral sound localization but also in SiN perception (22, 23). Previously, we found that the cortical activity patterns evoked by the sound localization paradigm differ between left- and right-sided deafness (24). Indeed, the outcomes from previous studies suggest that sound-in-noise processing is different depending on the side of deafness. For instance, it has been reported that unlike right-sided deafness, left-sided deafness is accompanied by behavioral advantages for cognitively demanding sound localization and SiN tasks, which are likely related to higher brain functioning due to intact contralateral projection from the peripheral to the central auditory system (25). Vannson et al. (25) suggested an association between sound localization and cortical functional activity; they found that localization ability was better in participants with left-sided deafness than those with right-sided deafness and behavioral performance was related to stronger brain activation. The increased cortical activity in left-sided deafened people was assumed to be compensation for the loss of binaural hearing (26, 27). On the other hand, poorer localization performance was revealed by the right-sided deaf group, which was associated with larger activity ipsilateral to the hearing side. Moreover, prolonged reaction time to locate sound sources in the horizontal plane in right-sided deafened people offers more evidence for the functional difference with left-sided deafness, which can be interpreted as the consequence of the longer processing time needed to reach the right hemisphere in which auditory spatial cues are predominantly processed (24).

Apparent localization deficit based on auditory spatial perception after damaging areas in the auditory cortex in humans and animals, respectively, is distinctly different. In animal studies, the ability to locate sound sources on the opposite side to the damaged hemisphere is considerably decreased regardless of the ablated side (28). In contrast, damage to the right hemisphere in humans has a more pronounced effect on the ability to localize sound than damage to the left one. Zatorre and Penhune (29) suggested that damage to the right auditory cortex can disrupt spatial perception on both sides. Furthermore, it has been reported that patients with right hemisphere damage have significantly impaired sound localization from any location whereas those with left hemisphere damage are capable of locating sounds from the ipsilateral hemispace (29–31). Thus, it can be inferred that the auditory cortex in humans plays the role in supporting spatial processing and behavioral localization, which is in contrast to animals in which many aspects of sound localization can be accounted for by neural processing at the subcortical level. To determine whether unilaterally driven plasticity is different depending on the side of deafness, we compared the pattern of neural activity between left- and right-sided deafness at the cortex level in the present study.

In the current study, we measured cortical N1/P2 responses because these components are thought to be related to sensory encoding and cognitive processes, including SiN listening (32). An auditory cortical evoked response is known to be sensitive to the features of the stimulus, such as its intensity and frequency (33). Given that SiN perception relies on both accurate sensory encoding and successful cognitive processing, we expect that the N1/P2 responses are related to behavioral SiN ability in SSD people. In NH listeners, substantial changes in hemispheric lateralization for SiN tasks have been observed in that functional asymmetry shifts from the right to left hemisphere during adverse listening conditions (6). However, there is still uncertainty as to whether the rightward activation for SiN perception is consistently shown by persons with SSD. Since alteration of the functional lateralization following monaural hearing deprivation is different depending on the side of deafness (34–36), we anticipated that the SiN-induced changes in cortical activation and hemispheric laterality are distinct for left-sided and right-sided deafness. Furthermore, consistent with previous reports, we hypothesized that cortical activation is weaker and temporally prolonged as the duration of deafness becomes longer (24, 37).

## Methods

### Participants

Ten adults with right SSD (RSD; 6 female, mean age: 52.7 ± 6.2 years) and 10 with left SSD (LSD; 6 female, mean age: 41.9 ± 16.8 years) were recruited. All of the unilaterally deaf participants were right-handed and had profound hearing loss in one ear (average pure-tone audiometry threshold >90 dB HL) and NH (pure-tone thresholds <20 dB HL from 0.25 to 4 kHz, with evoked otoacoustic emissions) in the other ear. Neither of the unilaterally deaf groups had used a hearing aid before participating in this study. Eleven age- and gender-matched NH adults were recruited for comparison with the SSD groups (NH, 7 female, mean age: 52.2 ± 6.9 years). The NH group participants had normal pure-tone average thresholds in both ears and no neurological and cognitive issues. Informed consent was obtained from all participants prior to testing. All experimental protocols and methods were approved by the guidelines and regulations outlined in the Sacred Heart Hospital of Hallym University Institutional Review Board (IRB no. 2019-02-019) and were performed in accordance with the ethical standards laid down in the 1964 Declaration of Helsinki. A summary of the clinical data of participants with SSD is provided in Table 1.

**Table 1:** Clinical features of participants with single-sided deafness.

**Participant**	**Deaf side**	**Age (year)**	**Gender**	**Duration of deafness (year)**	**Deafness onset (year)**	**Etiology**	**DE PTA threshold (dB HL)**	**NHE PTA thresholds (dB HL)**
1	Rt	55	F	49	6	Unknown	118	10
2	Lt	76	M	7	69	ISSHL	118	11
3	Rt	64	F	6	58	ISSHL	117	13
4	Rt	58	M	3	55	Shock	83	6
5	Lt	45	M	37	8	ISSHL	87	17
6	Lt	50	F	11	39	Virus	86	6
7	Lt	42	F	34	8	Unknown	98	13
8	Rt	57	F	3	54	ISSHL	111	17
9	Rt	46	F	13	33	Cholesteatoma	79	12
10	Rt	42	M	24	18	Shock	97	5
11	Lt	51	F	10	41	Meniere's disease	110	20
12	Lt	26	F	26	1	Unknown	117.00	0.00
13	Lt	44	F	29	18	Unknown	95	12
14	Lt	23	F	22	5	ISSHL	75.00	5.00
15	Lt	43	M	1	43	ISSHL		7
16	Lt	19	M	19	1	Unknown	93.00	12.00
17	Rt	57	M	1	58	ISSHL	104	11
18	Rt	48	F	33	17	Unknown	117.00	6.00
19	Rt	54	M	3	51	Noise-induced	81	3
20	Rt	51	F	5	46	ISSHL	117	0.00

PTA, averaged thresholds of 250, 500, 1,000, 2,000, and 4,000 Hz. ISSHL (Idiopathic sudden sensorineural hearing loss); LSSD, left-sided single-sided deafness; RSSD, rightt-sided single-sided deafness; DE, deaf ear; NHE, normal hearing ear.

### Stimuli and procedure

**Figure 1:**
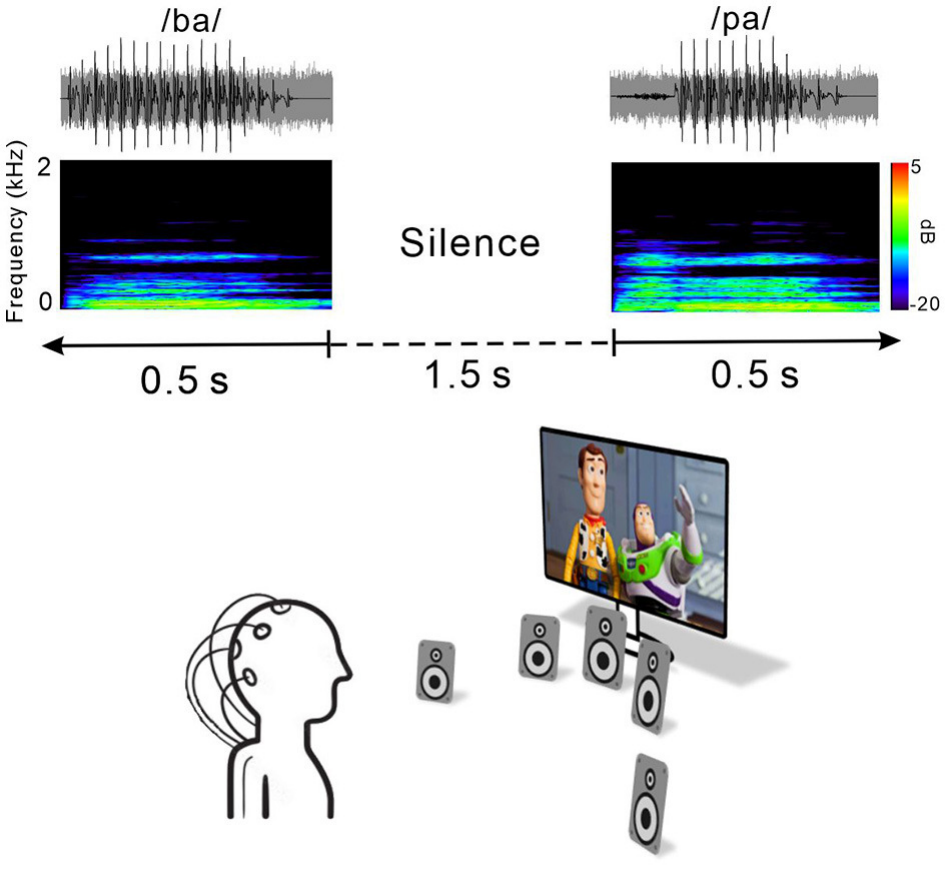
Speech stimulus and experimental listening conditions. Top, time waveforms of the CV syllable /ba/ and /pa/ with +5 dB speech-shaped masking noise. Middle, spectrogram of the acoustic stimuli. Each stimulus was embedded in 1.5 s of silence. Bottom, passive listening paradigm. During recording, subjects watched close-captioned movies of their choice.

Figure 1 shows an example of an acoustic sequence and the passive listening paradigm applied in this study. Natural /ba/–/pa/ speech stimuli with a noise masker at a signal to noise ratio (SNR) of +5 dB were used to evoke cortical responses. The noise masker was speech-shaped noise lasting 0.5 s created by applying the speech stimuli recorded from utterances by a male speaker and presented with speech stimuli simultaneously. The overall duration of each speech stimulus was 0.5 s, and the voice onset times were 30 and 100 ms for /ba/ and /pa/, respectively. The stimuli were presented through a StimTracker (Cedrus Corporation, CA, USA) system that allowed for EEG synchronization with the sound, and they were calibrated using a Brüel and Kjær (2260 Investigator, Nærum, Denmark) sound level meter set for frequency and slow time weighting with a ½ inch free-field microphone.

For each electrophysiological test, speech stimuli /ba/ and /pa/ were presented through a loudspeaker horizontally located at each of five different azimuth angles (−60°, −15°, 0°, +15°, and +60°, where “+” indicates the right side while “–” indicates the left side) under both quiet and noise listening. The stimuli were randomly presented with an inter-stimulus interval from sound offset to onset fixed at 1.5 s. A total of 1,000 trials involving 100 trials each for the /ba/ and /pa/ sounds at the five different azimuth angles under quiet and noise listening conditions (ba/pa x five azimuth angles x quiet/noise conditions) were presented across two blocks. During recording, the subject was seated in a comfortable reclining chair and watched a silent closed-captioned movie of their choice while the stimuli were individually presented in the background through a loudspeaker horizontally located at each of the five different azimuth angles. The subject was instructed to ignore the sounds and not to move their head during the experiment. During the recording, the subject was alert and calm.

All of the speakers were located 1.2 m away from the subject at ear level and sounds were presented at 70 dB SPL (sound pressure level). Breaks were given upon request. The total recording time was ~40 min.

### Behavioral tests

All subjects including the SSD patients and NH controls participated in behavioral sound localization task. The sound localization was measured for speech sounds at the five different azimuth angles mentioned above. In each trial, speech stimuli were emanated from each speaker in a random order. For each of the presentation, participants indicate location where a sound was presented by pressing a corresponding button on the keyboard assigned a speaker number. For the task, stimuli were presented in 10 blocks of 1,000 trials (200 trials for each of the five different azimuth angles), with each lasting 4 min. Prior to undertaking the task, each participants completed 10 familiarization trials of the procedure. The sound localization task was conducted in a sound-attenuated booth. Speakers were 1.0 m from the subject's head. No feedback regarding the performance was given during the test. Only the sound localization test results for the behavioral performance are reported herein. Accuracy of sound localization task was calculated using the root-mean-square error (RMSE) and the mean absolute error (MAE). The RMSE was assessed using the root-mean square of the magnitudes of the differences between the azimuth of the sound location and the azimuth of the selected location across all trials. The MAE is the absolute error in degrees, divided by the total number of trials.

As a behavioral measure of speech perception, word-in-noise perception was measured by using the consonant perception test (CPT) (38). A total of 50 words were presented in a “C/V/C” (consonant/vowel/consonant) context with a female talker in speech-shaped noise at a SNR of 0 dB. The number of words correctly identified out of 50 was expressed as a percentage. Since the CPT is forced choice paradigm among 4 alternative choices, subjects were instructed to indicate which words were heard by choosing buttons *via* mouse click.

### EEG recording

Electrophysiological data were collected by using a 64-channel actiCHamp Brain Products recording system (Brain Products GmbH, Inc., Munich, Germany). An electrode cap was placed on the scalp with electrodes positioned at equidistant locations (39, 40). The reference channel was positioned at the vertex while the ground electrode was located on the midline 50% of the distance to the nasion. Continuous data were digitized at 1,000 Hz and stored for offline analysis.

### Data processing

Electrophysiological data were preprocessed by using Brain Vision Analyzer 2.0 (Brain Products GmbH, Inc., Munich, Germany). Data were band-pass-filtered (1–50 Hz) and down-sampled to 500 Hz. Visual inspection of the data included the removal of artifacts related to subject movements (exceeding 500 mV). Independent component analysis (ICA) (41) implemented in Brain Vision Analyzer was applied to remove artifacts related to eye blinking and movement, and cardiac activity. After ICA artifact reduction, the data were low-pass-filtered at 20 Hz and segmented from −200 to 1,000 ms with 0 ms at the onset of the stimulus and re-referenced to the average reference. Averages were obtained for each of the azimuth angles. Subsequent peak detection was performed by using the fronto-central electrodes for the N1/P2 components. Since we used an electrode cap with equidistant locations which use different electrode layout from the traditional 10–20 system, N1/P2 were measured from the averaged activities of three electrodes located at Cz in the international 10–20 system (40, 42).

### Source analysis

Auditory evoked potential sources were computed by using BESA Research 7.0 (Brain Electrical Source Analysis, GmbH, Germany) as described previously (43). Dipole source analysis for N1 activity was performed on individual averaged waveforms and was implemented by using an average head model. To measure the dipole source activity for each subject, two symmetric regional dipole sources were seeded in the region of the auditory cortex (Talairch coordinates: ±49.5, −17, 9). In the next step, dipole fitting was executed in the mean area over a 20 ms window around the N1 peak on the global field power. A goodness of fit (GOF) was assessed for each subject over the 20 ms window. Data revealing an 80% or lower GOF were excluded from further analysis. As a result, 9 RSD, 9 LSD, and 11 NH subjects showed 80% or greater GOFs. During the analysis, the dipole sources were varied in location, orientation, and strength to fit tangential sources at the activation period maxima. The mean current over the 20 ms window centered on the peak of the tangential sources were assessed to conduct statistical analysis in each subject. In addition, BESA statistics 2.0 was performed for source space analysis. For the analysis, data files were created to compare between conditions (e.g., SiQ vs. SiN). The data files included information regarding source modeling in a 20 ms window in which maximal peaks were observed in the global field power. For the source modeling, sLORETA (standardized low resolution brain electromagnetic tomography) was conducted to evaluate source activation of individual subjects in the time range from 0 to 500 ms after stimulus onsets. The source activation differences in source space between SiQ and SiN conditions were assessed for each subject group using a paried *t*-test.

### Statistical analysis

Repeated-measures analysis of variance (ANOVA) was performed for the behavioral data to examine the effects of noise (SiQ vs. SiN) and subject group (NH, RSD, and LSD) on the RMSE and MAE. The repeated-measures ANOVA was also conducted to assess the effects of azimuth angle, noise, and subject group on amplitudes and latencies of N1/P2 cortical potentials. For comparing brain activity during SiN and SiQ listening, we used the SiQ data presented in our previous study (24). Tukey's Honest Significant Difference (HSD) test was conducted for *post hoc* comparisons, while Pearson product-moment correlations were used to assess correlations between the behavioral/audiological data and the N1/P2 activities for the SSD groups. For the dipole source data, group differences in hemispheric laterality were calculated by using grand mean source waveforms. In addition, paired *t*-tests corrected for multiple comparisons and Monte-Carlo resampling techniques implemented in BESA Statistics 2.0 (44) examined differences in the strengths of the brain source spaces between the listening conditions. Clusters of voxels with *p*-values of < 0.05 were considered significant, and the alpha criterion was manually set to 0.05 in BESA.

## Results

### Behavioral sound localization

**Figure 2:**
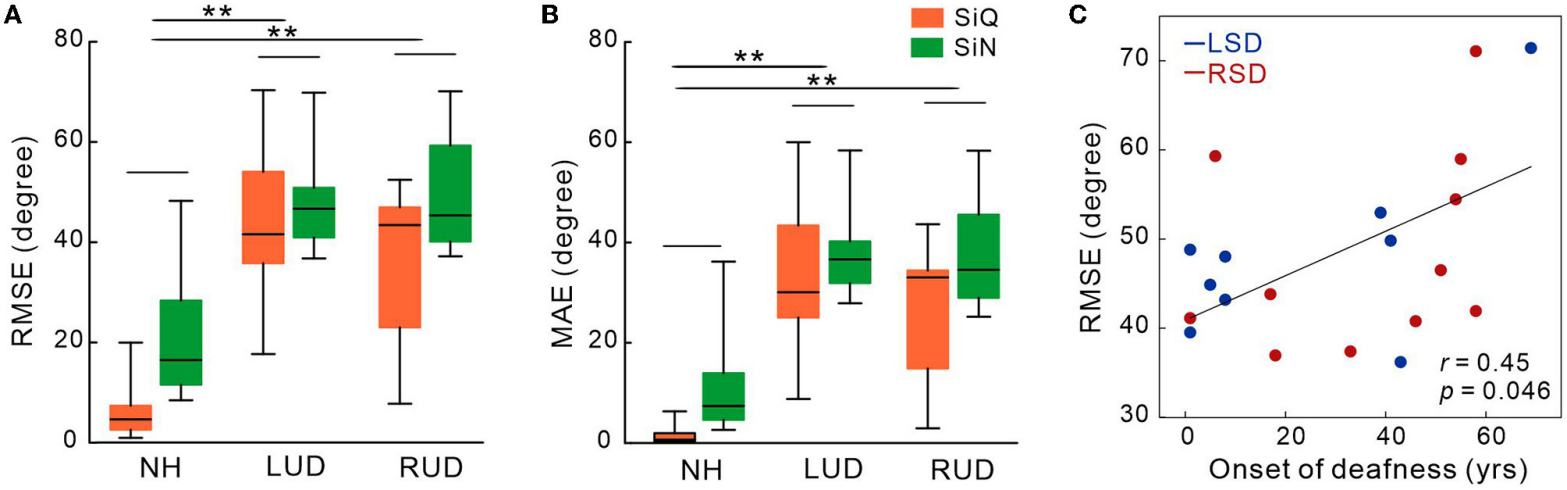
Mean root-mean-square error **(A)** and mean absolute error in degree **(B)** in subject groups with normal-hearing and with left- and right-sided deafness during the speech-in-quiet and speech-in-noise listening conditions. **(C)** The root-mean-square error correlations with the onset of deafness in SSD groups. Error bars: the standard error of the mean. RSD, right-sided single-sided deafness; LSD, left-sided single-sided deafness; NH, normal hearing; SiN, speech-in-noise; SiQ, speech-in-quiet; RMES, root-mean-square error; MAE, mean absolute error. **P < 0.01.

Figures 2A, B show the mean RMSE and MAE for each subject groups. Repeated-measures ANOVA analyzing RMSE data revealed significant effects of noise [*F*(_1,27_) = 19.3; *p* < 0.0001] and group [*F*(_1,27_) = 30.47; *p* < 0.0001]. Tukey's HSD *post hoc* tests revealed the RMSE was larger (worse) in both LSD (*p* = 0.001) and RSD (*p* = 0.001) groups than in NH group. No difference in the RMSE was found between LSD and RSD groups. For noise effect, the RMSE was smaller for the SiQ than for the SiN condition (*p* = 0.001). Similar to the RMSE, significant group [*F*(_1,27_) = 29.9; *p* < 0.0001] and noise [*F*(_1,27_) = 20.1; *p* = 0.0001] effects were found for the MAE. *Post hoc* tests conducted for the group effect showed the MAE was larger in the SSD groups (both *p* = 0.0001) and SiN (*p* = 0.0002) compared to NH group and the SiQ condition, respectively. Figure 2C shows a correlation between the RMSE and age at the onset of deafness in SSD groups. The results indicate that the RMSE was greater as the age at the onset of deafness is older (*r* = 0.45, *p* = 0.046).

**Figure 3:**
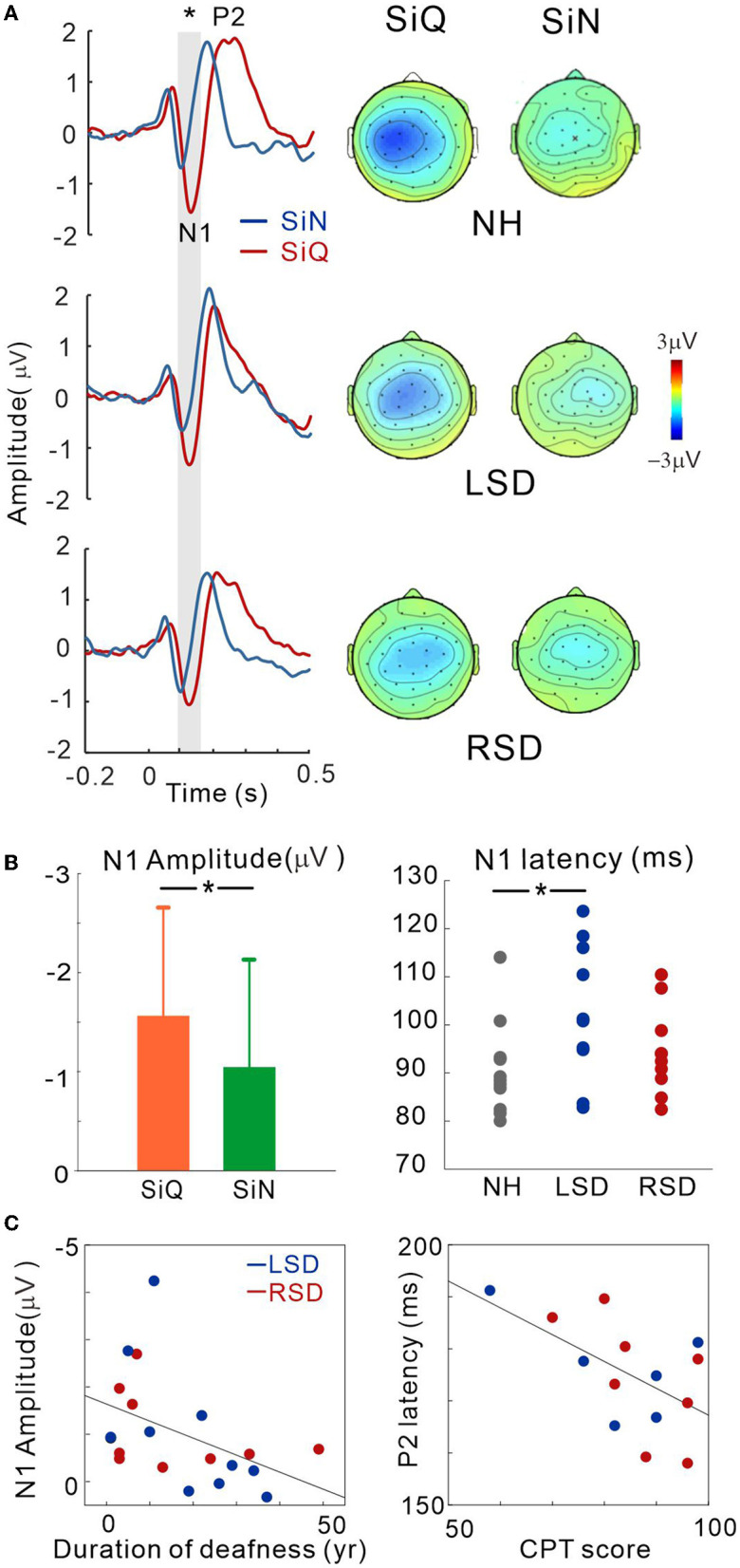
**(A)** Grand mean waveforms averaged stimuli emanated at azimuth angles of ±60° for the NH and ipsilateral to the hearing side for the SSD groups (+60° for the LSD; −60° for RSD) recorded *via* the frontal central (FC) electrodes for each subject group. Event-related potentials are shown for SiN (blue) and SiQ (red) listening. The gray highlighted area indicates the time window to measure the N1 response (80–150 ms). Topographical representation of the N1 response is presented for each subject group on the right side. **(B)** Listening condition (averaged across all groups) and group (averaged across all angles) comparisons for N1 amplitude and N1 latency, respectively. The error bars represent the standard error of the mean. *P < 0.05. **(C)** N1 amplitude correlations with the duration of deafness, and P2 latency correlation with word-in-noise perception scores measured using the CPT. RSD, right-sided single-sided deafness; LSD, left-sided single-sided deafness; NH, normal hearing; SiN, speech-in-noise; SiQ, speech-in-quiet.

A subset of SSD subjects was able to complete the CPT tests, the results for which are shown in Figure 3C (the right panel). The average scores for CPT were 82.3 for left-sided deafness and 86.7 for right-sided deafness. The results of an independent samples *t*-test revealed no significant difference between the test scores (*p* > 0.05).

### Cortical potentials

Figure 3A shows the grand mean waveforms for stimuli emanated at azimuth angles of ±60° for the NH and ipsilateral to the hearing side for the SSD groups (+60° for the LSD; −60° for RSD) under both SiN and SiQ conditions. The overall response was characterized by an N1 evocation at around 100 ms after stimulus onset followed by a P2 response. The magnitude of N1 was greater for SiQ than SiN listening whereas the P2 magnitudes were similar. Topographic examination of the N1 responses indicates that negativity was stronger for SiQ than SiN listening for all of the groups, while N1 activation in the NH group was lateralized toward the left hemisphere but more symmetrical over the brain in the SSD groups (Figure 3A).

Repeated-measures ANOVA was applied to examine the effect of sound location (−60°, −15°, 0°, +15°, +60° azimuth angles), type of stimulus (SiQ and SiN), and the group effect (NH, RSD, and LSD) on N1/P2 measures. Figure 3B shows the N1 amplitudes for the SiQ and SiN condition (averaged across all subjects) and N1 latencies for each subject groups (averaged across all angles). A significant effect of noise [*F*(_1,27_) = 5.7; *p* = 0.024] was found for N1 amplitude. Tukey's HSD *post hoc* test results show that the N1 amplitudes for SiQ were larger than those for SiN (*p* < 0.01). In addition, a significant group × angle interaction [*F*(_8,108_) = 2.58; *p* = 0.012] was revealed, and Tukey's HSD *post hoc* test results reveal that in the LSD group, the N1 amplitudes at azimuth angles of 0° (*p* = 0.043), +15° (*p* = 0.034), and +60° (*p* = 0.007) were smaller than those at −15°. In the RSD group, the N1 amplitudes at an azimuth angle of +60° were larger than those at −15° (*p* = 0.037) and +15° (*p* = 0.034). No significant interaction was found in NH group. For N1 latency, a significant effect of group [*F*(_2,28_) = 3.66; *p* = 0.038] was found, and *post hoc* test results indicate that the N1 latencies for the LSD group were longer than those for the NH group (*p* = 0.011).

No significant differences were found for P2 amplitude. However, a significant effect of azimuth angle [*F*(_4,184_) = 4.5; *p* = 0.002] was found for P2 latency. Tukey's HSD *post hoc* test results show that the P2 latencies at azimuth angles of −60° and +60° were longer than those at −15° (*p* = 0.001 for −60° and *p* = 0.011 for +60°), 0° (*p* < 0.001 for both), and +15° (*p* = 0.009 for −60° and *p* = 0.017 for +60°).

To assess whether N1/P2 responses to SiN stimuli are related to audiological factors or behavioral speech perception in SSD subjects, we examined the relationships between averaged N1/P2 measurements according to the duration of deafness and CPT scores. Since not all of the SSD subjects provided CPT scores, data from only 14 subjects (6 and 8 from the LSD and RSD groups, respectively) were used in the correlation analysis. Figure 3C shows that the averaged N1 amplitudes across all azimuth angles during the SiN task were inversely related to the duration of deafness in the SSD groups (*r* = −0.45, *p* = 0.047), suggesting that N1 decreases with a longer duration of deafness. In addition, the averaged P2 latencies were negatively correlated with CPT scores, indicating that P2 latency is shorter with better word-in-noise performance (*r* = −0.57, *p* = 0.034).

### Dipole source analysis

**Figure 4:**
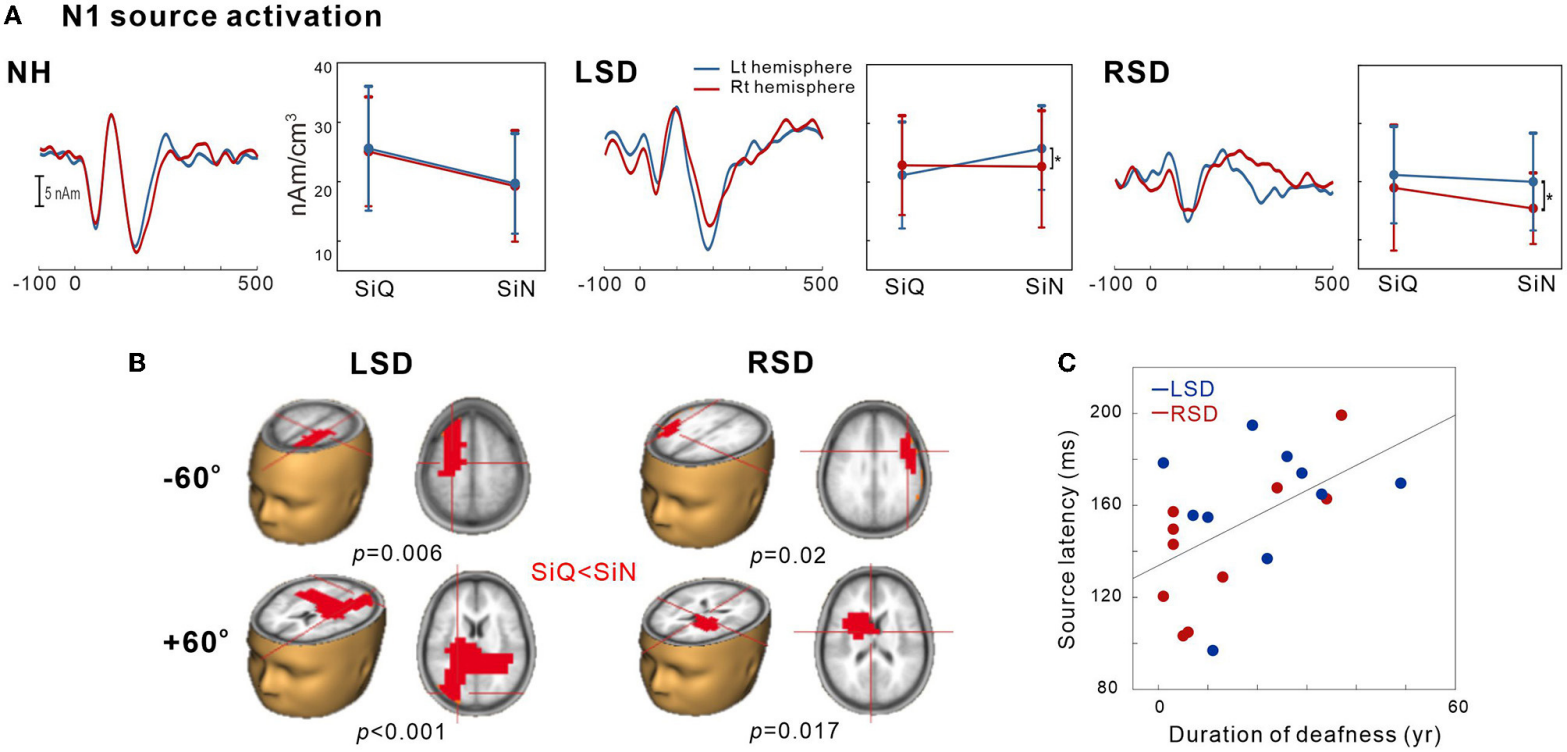
N1 source activation by the speech-in-quiet (SiQ) and speech-in-noise (SiN) stimuli. **(A)** The grand mean N1 dipole source waveforms for the left (blue) and the right hemispheres (red) for each subject group. The mean N1 source amplitudes for SiQ and SiN listening are presented for each group on the right side of the mean waveforms. The error bars represent the standard error of the mean. **(B)** Cluster data representing significant differences between the SiQ and SiN listening tasks in the brain source space. Red indicates that the SiN listening response was larger than for SiQ (a positive difference). Note that these clusters indicate which regions showed a significant difference while the crosshairs indicate a 3D point indicating the maximum difference in the comparison. **(C)** N1 source latency correlation with duration of deafness. RSD, right-sided single-sided deafness; LSD, left-sided single-sided deafness; NH, normal hearing.

This was conducted to examine the tangential source of N1 for SiN perception. To measure the SiN effect on N1 source activation, we also assessed the tangential components while SiQ listening and then compared them while SiN listening. Figure 4A shows N1 source activation averaged across all of the azimuth angles for the left and right hemispheres of the NH, LSD, and RSD groups. Repeated-measures ANOVA was conducted to measure the effects of noise, azimuth angle, and subject group on N1 dipole source amplitude and latency. For N1 source amplitude, a significant group/hemisphere interaction [*F*(_2,28_) = 8.25; *p* = 0.001] was found. Tukey's *post hoc* analysis revealed that during SiN listening, N1 source activation in the LSD group was greater in the left hemisphere, which is contralateral to the hearing side (*p* = 0.029), while stronger ipsilateral activation (left hemisphere) was found in the RSD group (*p* = 0.002). No statistically significant difference between the hemispheres was found for SiQ listening, and no statistically significant asymmetrical dipole activation was found in the hemispheres of the NH group participants (both *p* > 0.05).

Figure 4B shows the *t*-test comparisons for the N1 source space to compare between the SiQ and SiN conditions for +60° and −60° azimuth angles in SSD groups. For this comparisons, we focused on −60° vs. +60° for the following reasons: (1) the findings in previous electrophysiological data suggest that N1 cortical activity is larger for stimuli containing more prominent spatial cues than for less spatially distinguishable stimuli (45); and (2) given that the −60° and 60° azimuth angles are closer to the hearing and deafened ears than the other angles, these conditions could better represent the effect of SSD on source activation. (3) cortical N1/P2 responses to ± 60° in SSD subjects were more delayed and larger compared to the smaller azimuth angles. For the LSD group, comparisons between SiQ and SiN listening revealed significant clusters (*p* = 0.006) with stronger activation in the frontal lobe (the left premotor and supplemotor areas) during SiN listening at an azimuth angle of −60° and in the temporal and occipital lobes at +60° (*p* < 0.001). Meanwhile, for the RSD group, significantly larger activation was found in the frontal area during SiN listening at azimuth angles of −60° (*p* = 0.02) and +60° (*p* = 0.017) (the left Broca area at −60° and the left dorsal anterior cingulate cortex at +60°).

Figure 4C depicts relationships between averaged N1 source latency and duration of deafness in subjects with SSD. Pearson product-moment correlation results revealed that N1 source latency to SiN stimuli was positively correlated to the duration of deafness (*r* = 0.53, *p* = 0.017), indicating more prolonged latencies with the longer duration of deafness.

## Discussion

The aim of this study was to compare cortical responses during SiQ and SiN listening to characterize the cortical representation of SiN processing in persons with SSD. Given that the distinct patterns of brain activation depending on the side of deafness have been reported (24, 35, 46), we also compared the neural responses in LSD and RSD participants during SiN listening. We found that (1) the SiN is differently processed in the brains of left- and right-sided SSD in that the left-sided SSD revealed greater activity contralateral to the hearing side (left hemisphere), while the right-sided SSD showed the left hemispheric asymmetry. (2) the N1 modulation as a function of sound location was more evident in participants with left-sided deafness. In addition, (3) N1 activity and sound localization performance in SSD participants were associated with the deafness duration and the onset of deafness, respectively.

Analysis of N1 dipole source activity revealed that noise-degraded speech sounds incur differential effects on the hemispheric laterality depending on the side of deafness. For SiN listening, activity contralateral to the hearing side was greater with left-sided deafness but a contrastive pattern of lateralization in that stronger ipsilateral bias was revealed with right-sided deafness. Interestingly, no hemispheric laterality was found for SiQ listening in either SSD group. These results in dipole source activation enabled us to tease out the contributions of the hemispheres engaged in processing SiN stimuli and to confirm that the auditory system has active compensatory mechanisms mitigating degraded speech processing. In listeners with NH, contralateral activity is more predominant for left- than right-ear stimulation (47, 48). Nonetheless, in persons with SSD, the adaptation process of the left hemisphere could strengthen both the ipsilateral and contralateral pathways for processing degraded speech sounds. With right-sided deafness, stronger left-hemispheric activity has been attributed to functional plastic changes mainly occurring in the left hemisphere rather than the right one (49, 50). Strengthening the routes to the left hemisphere for SiN listening could be related to the left hemisphere playing a crucial role in speech and language function (51), which mainly contributes to the right-ear advantage for processing degraded speech sounds (16).

The results of a previous study examining alpha and theta rhythms in children with unilateral deafness and NH controls are inconsistent with our findings in that the neural activity in the NH group was lateralized to the left side during a quiet listening task whereas rightward asymmetry was found during a word-in-noise recognition task (21). However, this pattern of asymmetry decreased in children with unilateral hearing loss due to attenuated asymmetrical activation (21). Possible explanations for this discrepancy are (i) the type of noise used for evoking the response, (ii) the subjects' characteristics, and (iii) the listening conditions (passive vs. active). Given that for NH, increasing the SNR decreases lateralization toward the right hemisphere (32), the low SNR used in our study would weaken hemispheric lateralization in NH listeners. In addition, most of the SSD participants in our study were adults who had acquired auditory deprivation later in life whereas children with congenital unilateral hearing loss were mainly recruited for the previous study by Cartocci et al. (21). It has been demonstrated that asymmetrical hearing loss occurring during early childhood compromises brain lateralization due to incomplete auditory development (52). In this regard, Burton et al. (49) proposed that congenital unilateral deafness can result in strengthening the contralateral pathway while acquired unilateral deafness can lead to over excitation of the ipsilateral pathway.

Interestingly, we found that cortical activation in right-sided deafness is independent of the direction of stimulation whereas left-sided deafness alters sites of activation according to the amount of spatial information. More specifically, in left-sided deafness, activation was found in the temporal and occipital lobes when sounds were presented from the side of the intact ear (an azimuth angle of +60°), while the activation was greater in the frontal lobe for the stimuli presented on the deaf side (an azimuth angle of −60°). On the other hand, right-sided deafness produced strong activity in the frontal areas regardless of the side of stimulation (Figure 3B). The differential recruitment of the frontal and temporal regions for encoding spatial information could be closely related to the functional change brought about by cortical reorganization according to the side of deafness. The neural generators contributing to processing speech under adverse listening conditions are located in both the frontal and temporal lobes: the temporal lobe is thought to play a role in initial sound processing while the frontal cortex is more associated with the higher-order speech processing such as SiN listening (53). Indeed, the extensive frontal-temporal network including the anterior cingulate and the prefrontal cortex are preferentially activated for processing linguistic and spatial information (9, 54–56). However, in individuals with SSD, such functional organization of the cortex for SiN processing seems to be altered by deafness-driven plasticity. In particular, the activation of the frontal cortex observed in persons with SSD could reflect active adaptation processing in the cortex to enable higher cognitive resources to process degraded speech stimuli. Considering that individuals with right-sided deafness show the frontal lobe activation required to process sounds from both the deaf and hearing sides, right-sided deafness could require more effort for SiN processing than left-sided deafness in which the activation sites are allocated according to the side of stimulation. This interpretation is supported by a neuroimaging study showing that right-sided deafness is related to higher activation of the frontal cortical regions not seen in persons with left-sided deafness (57); the authors concluded that right-sided deafness enhances activation in the areas involved in the processing of degraded sounds. Our data corroborate this finding by explicating differential reorganization of the cortex according to the side of deafness for processing impoverished speech stimuli.

In addition to the different activation patterns between left- and right-side deafness, it is important to note that the roles of peripheral and central processing deficits in SiN perception differ between bilateral and unilateral deafness. Although both types of hearing loss induce changes in the central auditory system, peripheral loss is totally different. Bilateral hearing loss is accompanied by an elevation in the hearing threshold and a decrease in spectral processing but with access to bilateral cues in both the intensity and timing domains (58). On the other hand, hearing *via* the good ear in unilaterally deafened individuals can be as good as that of normal listeners for SiN perception when the speech and noise are presented to the good ear. In this sense, the loss of sound source localization is the main issue for SSD subjects in the case of a single talker whereas the loss of binaural processing reflects true SiN perception in the presence of multiple talkers.

Binaural processing is important for both sound localization and SiN perception because the neural processing for both tasks is closely related to each other (59). In the cocktail party situation, binaural hearing helps to lessen the masking of the target sounds by noise presented from other directions. Based on this effect, the binaural masking level difference (BMLD) improves sound detection when the phase of either the signal or the noise is inverted (60). In a free-field environment, a similar level of unmasking in humans (61) and animals (62) is obtained by spatially separating the signal and the masker. In an animal study, it has been found that the responses of the inferior colliculus (IC) neurons to BMLD stimuli are consistent with their ITD sensitivity to tone and noise (63). Furthermore, behavioral and functional changes with unilateral deprivation have been reported in animals with SSD. In particular, the outcomes of several single-neuron studies on the effect of unilateral deafness at the brainstem and cortex levels suggest an increase in the responsiveness of the IC and the primary auditory cortex neurons to acoustic stimulation on the side of the intact ear (64–66). For example, unilateral hearing loss in barn owls was accompanied by compensatory shifts in ITD sensitivity at the IC level (67). This may be related to weakening of the auditory pathways that convey input from the deprived ear in several brain areas, including the cochlear nucleus (68), the superior olive (69), and the IC (70). This outcome indicates that a change in the auditory pathway affects the capacity of the auditory system to adapt to unilateral deafness by becoming more dependent on the monaural spatial cues provided by the hearing ear.

At the neuroanatomical level, neurons in the IC change substantially following unilateral hearing loss because they need to be able to integrate various auditory spatial cues (71). In turn, it can be inferred that the IC is more susceptible to brain plasticity than other auditory pathway sites due to its functional characteristics (72). In animal studies, unilateral hearing loss weakened ipsilaterally mediated suppression in the IC ipsilateral to the deprived ear, albeit not at the level of the auditory cortex (73). In this respect, these results indicate that neuronal changes following unilateral deprivation are more apparent at the subcortical level rather than at the cortical level. Nonetheless, the neural basis for unilaterally deafened-induced plasticity at the IC level is not well characterized in humans, thereby suggesting the need for future work in this area.

Concerning the relative roles of peripheral and central processing deficits in SiN recognition by individuals with SSD, one important factor for SiN processing is the acoustic properties of the noise maker. The sensory aspects of SiN can be considered as how the acoustic signal is transduced by the ear and transmitted and transformed along the central auditory system. External noise can cause disruption in signal encoding in the central auditory system, and for this reason, it is frequently referred to as a masker. Meanwhile, noise that interacts with a signal, leading to a degraded neural representation, is generally referred to as an “energetic masker.” The term “energetic” comes from the level of interaction between the masker and the signal within the same critical bands at the same time. On the other hand, “informational masking” consists of a masker that is outside the critical bands so that both the target signal and the masker are audible. Energetic masking can produce interference within the peripheral auditory system whereas informational masking is often taxing on the cognitive resources required for selective attention (74). Given that the SiN performance of bilaterally or unilaterally deafened individuals according to the type of noise can vary, the effects of noise type and spatial cues on the performance have been extensively studied. In people with SSD, better performances were obtained with single-talker noise compared to using a multiple-talker distractor (75). In addition, SSD subjects perform poorly when speech and noise are presented from the same speaker due to a reduction in spatial cues (76). Meanwhile, listeners with bilateral hearing are more affected by multi-talker noise due to the loss of binaural hearing (3). Acoustically, multi-talker noise is dominated by energetic masking while single-talker noise contains both energetic and informational masking. Therefore, it can be inferred that single-talker noise with informational masking is more difficult for SSD individuals since it requires more attentional cognitive resources. Furthermore, this supports the notion that the cortical plasticity following monaural deprivation may not enhance some aspects of binaural hearing involving informational masking.

Under normal circumstances, N1/P2 cortical activities in response to acoustic noise decrease in amplitude and increase in latency (15, 32, 77). Similar to the observations for NH listeners, cortical N1 responses in persons with SSD were smaller during SiN compared to SiQ listening (36). However, our data reveal that in SSD individuals, the effect of degraded speech sounds on P2 response is much smaller than on N1 response. These results expand on previously reported findings by suggesting that noise-related changes can be mainly attributed to the N1 components. Our findings indicate that N1/P2 activities in persons with SSD undergo distinct changes with noise. It is known that N1 is significantly affected by the stimulus characteristics, such as frequency (78), intensity (79), and acoustic changes (40), whereas P2 is related to more higher-order cognitive processing, including perceptual experience (80) and auditory training (81, 82). When processing degraded speech sounds, N1 relies solely on the SNR without taking acoustic properties such as the absolute intensity of the signal into consideration (77, 83). This notion is supported by the findings from a previous study comparing responses to tone bursts with various levels of background noise in which substantial changes in the N1 amplitude as a function of noise were observed while no effect was evident for intensity changes (83). Contrary to N1 showing consistent changes with the noise masker, P2 noise-related changes are largely variable. Papesh et al. (84) reported that P2 is affected by interactions among stimulus variables including signal type, noise type, and experimental paradigm. Therefore, we assume that our experimental design is suitable for inducing changes in neural generators underlying the N1 rather than the P2 response.

Our results concerning the relationship between N1/P2 cortical activities and behavioral performance in SiN perception are in agreement with those from previous event-related potential studies (21, 24, 36). Notably, we found that sensor-level N1 amplitude and P2 latency are associated with the duration of deafness and word-in-noise ability, respectively. At the source level, N1 activity can be used to predict the duration of deafness and subjective speech perception in persons with SSD. In other words, the N1 response becomes progressively weaker with decreasing SiN perception and a longer duration of deafness. These results suggest that the brain mechanisms required for the neural processing of SiN stimuli are more difficult to induce in SSD individuals with longer duration of deafness. Given that a positive correlation between N1 activity and behavioral/perceptual SiN ability has been observed, successful SiN perception in persons with SSD could require more faithful neural encoding of degraded auditory input at the cortical level. However, the quality and amount of the brain plasticity in some SSD individuals (i.e., chronic SSD) is not sufficient to improve the neural activity for robust SiN perception (85, 86). In this case, higher-order cognitive controls such as attention and memory might efficiently improve SiN listening in SSD individuals (20). Taken together, our data leads us to infer that chronic unilaterally deafened people develop “SSD-specific” neural mechanisms to compensate for decreased ability to process SiN stimulus. Nevertheless, additional efforts to enhance cognitive controls such as auditory training could re-formulate the neural population required for SiN listening into a more “normal-like” pattern.

## Data availability statement

The raw data supporting the conclusions of this article will be made available by the authors, without undue reservation.

## Ethics statement

The studies involving human participants were reviewed and approved by Sacred Heart Hospital of Hallym University Institutional Review Board (IRB no. 2019-02-019). The patients/participants provided their written informed consent to participate in this study.

## Author contributions

J-HH and JL collected the data and analyzed the data. J-HH, JL, and H-JL contributed to writing the manuscript. All authors contributed to the article and approved the submitted version.
